# Supplementary material for: (-)-Epigallocatechin-3-Gallate Attenuates the Adverse Reactions Triggered by Selenium Nanoparticles without Compromising Their Suppressing Effect on Peritoneal Carcinomatosis in Mice Bearing Hepatocarcinoma 22 Cells
Source: Molecules. 2023 May 5;28(9):3904. doi: 10.3390/molecules28093904 (PMC10180376; doi:10.3390/molecules28093904)
Supplement: Supplementary file 1 [file molecules-28-03904-s001.zip › molecules-2377531-supplementary.pdf]

Supplementary materials

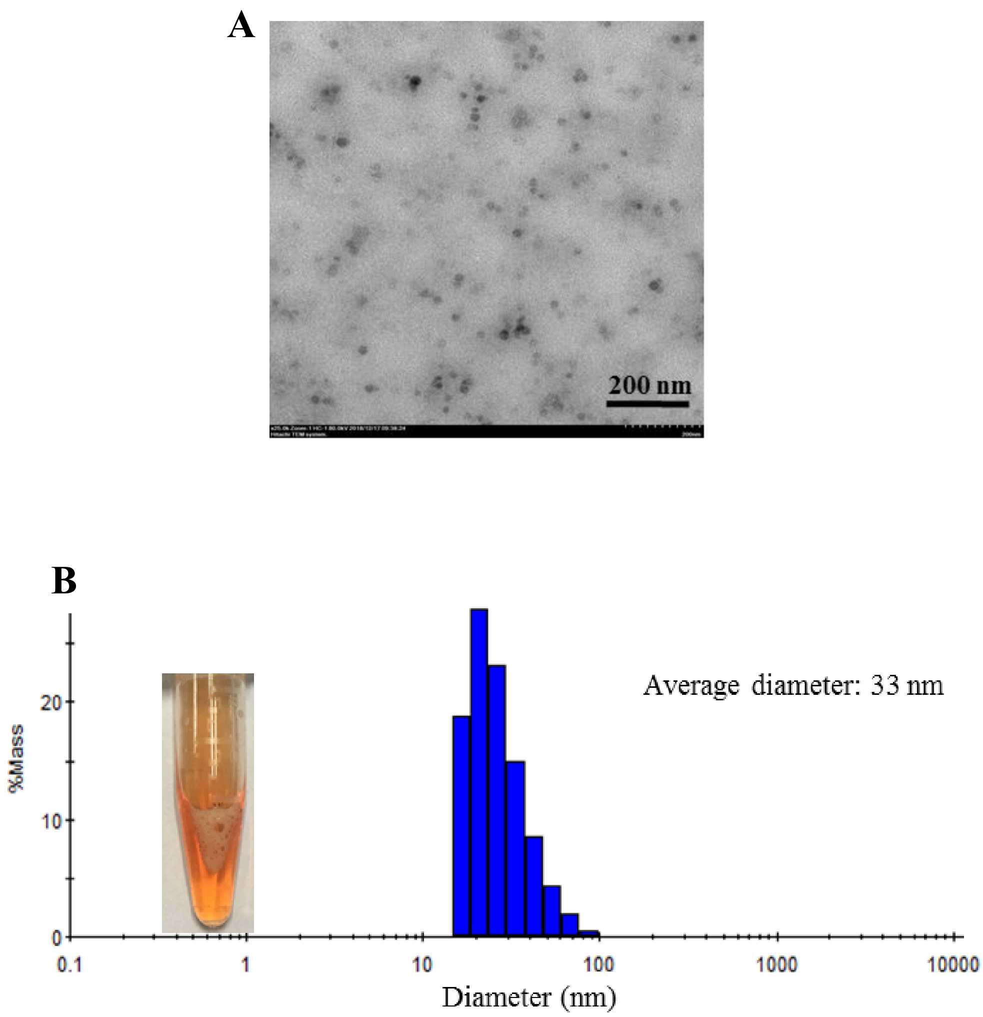

Figure S1. Characterization of SeNPs. (A) TEM. (B) Size distribution.

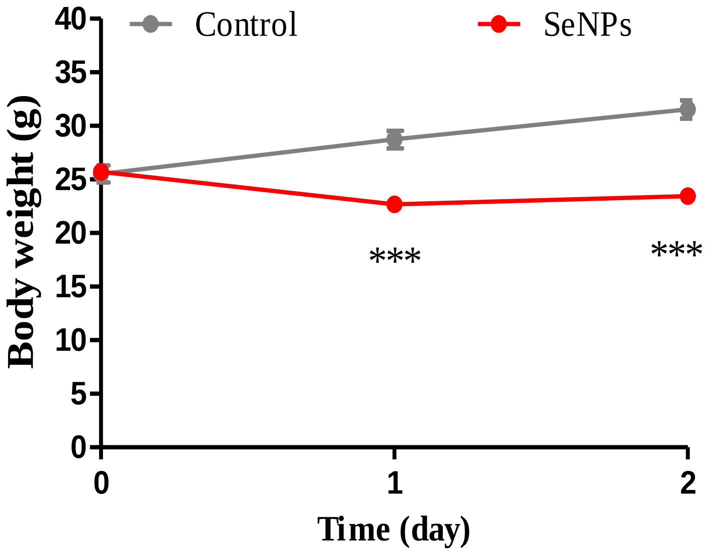

Figure S2. Body weight of mice post SeNPs treatment. **Experiment**: H22 model mice were i.p. injected with 3 mg/kg SeNPs, once.
